# Supplementary material for: The Discovery and Characterization of Conserved and Novel miRNAs in the Different Developmental Stages and Organs of Pikeperch (Sander lucioperca)
Source: Int J Mol Sci. 2023 Dec 22;25(1):189. doi: 10.3390/ijms25010189 (PMC10778745; doi:10.3390/ijms25010189)
Supplement: Supplementary file 1 [file ijms-25-00189-s001.zip › Table S6_Primers used in this study.pdf]

Table S6 Primers used in this study.

Mature miRNA	Forward primer
Tested reference miRNAs:	
slu-mir-26a-1-3-5p	TTCAAGTAATCCAGGATAGGCT
slu-mir-30-1-2-5p	TGTAAACATCCTTGACTGGAAGCT
slu-mir-30c-5p	TGTAAACATCCTACACTCTCAGCT
slu-mir-107-3p	AGCAGCATTGTACAGGGCTATC
miRNAs particularly abundant or differentially expressed in DESeq2:	
slu-mir-10b-5p	TACCCTGTAGAACCGAATGTGT
slu-mir-122-5p	TGGAGTGTGACAATGGTGTTTG
slu-mir-130a-5p	GTCCTTTTTCTGTTGCACTACT
slu-mir-130ab-3p	CAGTGCAATATTAAAAGGGCA
slu-mir-202-5p	TTCCTATGCATATACCTTTTTCAAAAAA
slu-mir-203b-3p	TGAAATGTTTAGGACCACTGA
slu-mir-205-5p	CTTCATTCCACCGGAGTCTG
slu-mir-551-3p	GCGACCCATCCTTAGTTTCTG
slu-mir-1388-3p	CTCAGGTTCGTCAGCCCATG
slu-mir-1388-5p	AGGACTGTCCAACCTGAGAATG
Hypoxia cluster:	
slu-mir-462-5p	TAACGGAACCCATAATGCAGCTG
slu-mir-731-5p	AATGACACGTTTTCTCCCGGATT
